# Retention of Knowledge Levels of Health Care Providers in Cancer Screening Through Telementoring

**DOI:** 10.1200/JGO.18.00048

**Published:** 2018-07-11

**Authors:** Roopa Hariprasad, Sanjeev Arora, Roshani Babu, Latha Sriram, Sarita Sardana, Sudarshan Hanumappa, Ravi Mehrotra

**Affiliations:** **Roopa Hariprasad**, **Roshani Babu**, **Latha Sriram**, **Sarita Sardana**, and **Ravi Mehrotra**, National Institute of Cancer Prevention and Research, Noida; **Sudarshan Hanumappa**, Karuna Trust, Bengaluru, India; and **Sanjeev Arora**, University of New Mexico, Albuquerque, NM.

## Abstract

**Purpose:**

Every year > 450,000 individuals are diagnosed with cancer and approximately 350,000 die of it in India. The Ministry of Health and Family Welfare has released an Operational Framework for the Management of Common Cancers that highlights population-based cancer screening programs in primary health care facilities by health care providers (HCPs) and capacity building of HCPs. The purpose of this study is to present a low-cost training model that is highly suitable for resource-deficient settings, such as those found in India, through Extension for Community Health Outcome (ECHO), a knowledge-sharing tool, to enable high-quality training of HCPs.

**Materials and Methods:**

An in-person, 3-day training program was conducted for 27 HCPs in the tribal primary health care center of Gumballi in Karnataka, India, to teach the basics of cancer screening in oral, breast, and cervical cancer. The training of HCPs was done using the ECHO platform while they implemented the cancer screening, thus enabling them to build the much needed knowledge and skill set to conduct cancer screening in their respective communities.

**Results:**

The knowledge level of the HCPs was tracked before the intervention, immediately after the 3-day training program, and 6 months after the ECHO intervention, which clearly showed progressive acquisition and retention of knowledge. A marked improvement in knowledge level score from an average of 6.3 to 13.7 on a 15-point scale was noticed after the initial in-person training. The average knowledge further increased to a score of 14.4 after 6 months as a result of training using the ECHO platform.

**Conclusion:**

ECHO is an affordable and effective model to train HCPs in cancer screening in a resource-constrained setting.

## INTRODUCTION

The three most commonly occurring cancers in India are those of the breast, uterine cervix, and lip or oral cavity, together accounting for approximately 34% of all cancers.^1^ Screening and early detection of these cancers will help to markedly reduce morbidity and mortality from these cancers. At present, screening in India is limited to sporadic opportunistic screening for malignancies of the cervix, breast, and oral cavity. The Ministry of Health and Family Welfare has recently (August 2016) launched operational guidelines for screening and prevention of the most common cancers.^2^ This population-based cancer screening program is going to be rolled out using the existing health care providers (HCPs) at the various facility levels of the Indian Public Health System.

Although there is an emphasis on prevention of noncommunicable diseases (NCDs), HCPs are not actively involved in NCD control, which also includes cancer. However, training HCPs can make them invaluable in implementing the cancer screening program and making gains toward the larger mission of NCD control in the nation.^3^ From past studies, we know that one-time training elevates the knowledge level of the attendees exponentially, but a substantial amount of knowledge is lost by 6 to 12 months after training.^4,5^ This highlights the need for repeat training programs to sustain the knowledge levels gained. Iterative training has been shown to help HCPs retain and use the knowledge obtained in training.^6^ However, such iterative training needs the HCPs to take time off their regular work and travel to district or state headquarters and is a financial drain on the health system.

Extension for Community Health Outcome (ECHO) is an online knowledge-sharing platform aimed at moving knowledge rather than moving patients or doctors. ECHO was started by Dr Sanjeev Arora to treat hepatitis C patients in rural areas of the state of New Mexico.^7,8^ Many ECHO projects are currently being conducted around the world for various diseases and health systems such as bone health,^9^ pain management, substance use disorder,^10^ and diabetes.^11^ The University of Texas MD Anderson Cancer Center has adapted the ECHO model to educate local providers in the management of cervical dysplasia in a low-resource region of Texas and to inform strategies for the management of advanced cervical and breast cancer in Latin America and sub-Saharan Africa.^12^ The goal of our study was to evaluate whether HCPs could sustain knowledge to implement population-based cancer screening.

## MATERIALS AND METHODS

The aim of this study was to evaluate the knowledge scores of HCPs before and after training in cancer screening and to determine the impact of the ECHO model on knowledge retention after 6 months of training.

### Study Area

This pilot study was conducted at the Gumballi primary health care center (PHC), located in Chamarajanagar in the state of Karnataka in southern India. The PHC caters to a population of approximately 22,000 people, which is composed predominantly of a rural population with an approximately 17% tribal population that is managed by the Karuna Trust, a nongovernmental organization.^13^

### Study Population

The HCPs at the PHC who underwent training were as follows: one medical officer, one dentist, three staff nurses, five auxiliary nurse midwives (ANMs), three male health workers, and 14 accredited social health activists workers. Their roles and responsibilities of these HCPs in implementing the cancer screening program are listed in Table 1.

**Table 1:**
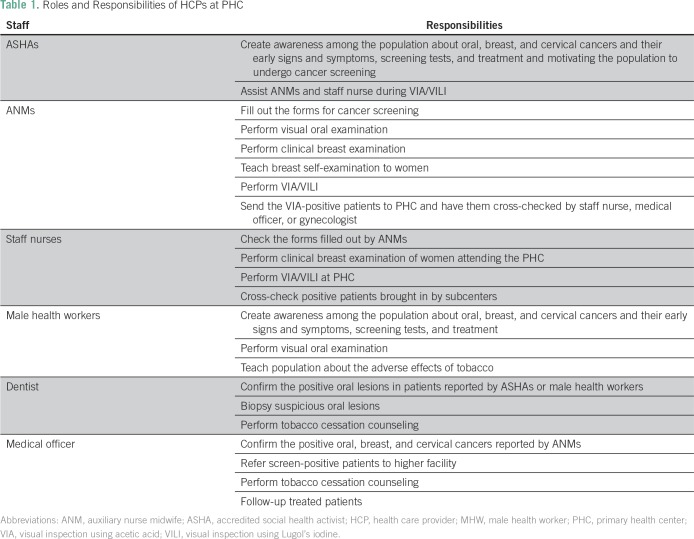
Roles and Responsibilities of HCPs at PHC

### Study Design

This is an interventional study of pre- and post-test design. This study was approved by the Institutional Ethics Committee at the National Institute of Cancer Prevention and Research (Indian Council of Medical Research).

### Study Questionnaire

This training session was evaluated using a self-administered questionnaire in vernacular language (Kannada) and was pretested in a small group of HCPs. The questionnaire consists of 15 multiple-choice questions divided into various sections covering all aspects of cancer including general information, such as risk factors and etiology, and in-depth knowledge about various forms of cancer (eg, oral, breast, cervical) and their symptoms, screening tests, detection methods, and treatment.

The same questionnaire was administered anonymously before and after training. It was administered again 6 months after implementation of the ECHO platform, after the completion of the initial training program.

### Intervention

The training was done by a trained gynecologist from the National Institute of Cancer Prevention and Research, in the vernacular language of Kannada, with which all of the HCPs were familiar.

#### Day 1.

At the start of the training program, a baseline evaluation of knowledge was conducted using the predesigned questionnaire. The training started with sharing knowledge about cancer screening (why, when, and how) and the purpose and objectives of cancer screening in the primary health care setting.

The following topics were discussed using PowerPoint (Microsoft, Redmond, WA) presentations along with pictures: normal anatomy and physiology of the vulva, vagina, and cervix; cervical cancer epidemiology, risk factors, and symptoms; human papillomavirus and cervical cancer; diagnostic tests for cervical cancer (ie, visual inspection with acetic acid [VIA], visual inspection with Lugol’s iodine, Papanicolaou test, and human papillomavirus testing); abnormal cervical lesions; management of screen-positive patients; negative patients to be requested to return for repeat screening after 3 years; protocol to refer screen-positive patients to tertiary cancer care facilities for further evaluation and treatment; knowledge of treatment modalities; and sterilization procedure for all the devices used in the process.

These topics were discussed along with hands-on demonstration of preparation of 5% acetic acid, the reagent used for the VIA test. The VIA test was also demonstrated to ANMs and staff nurses on women who attended the PHC. Because the visual impact of pictures is always better than just description, various pictures of different types of physiologic and pathologic cervical lesions were shown along with VIA-positive and -negative images for better understanding of diagnosis of cervical cancer. Throughout the training session, the trainees were encouraged to stop and clarify their doubts, as and when required. Toward the end of the training session for that day, many more queries raised by participants were clarified.

#### Day 2.

On day 2, breast and oral cancers were discussed. The following topics were discussed using PowerPoint presentations along with pictures: breast cancer epidemiology; risk factors and symptoms of breast cancer; clinical breast examination; breast self-examination; abnormal breast lesions; and knowledge of treatment modalities. Role play of counseling sessions was done, and afterward, there was a question-and-answer session.

Next, anatomy of the oral cavity, risk factors for oral cancer, and symptoms of oral cancer were explained. Some pictures of precancerous lesions were shown to the participants. The dentist posted at PHC explained detailed oral examination to all participants and demonstrated the examination on a patient who attended PHC. Treatment modalities of precancerous and cancerous lesions were explained.

#### Day 3.

On the final day, all of the topics discussed were reiterated, as follows. Oral, breast, and cervical cancer risk factors and symptoms, examination procedures, and counseling were reviewed. Images of different types of physiologic and pathologic cervical lesions were shown, and each participant was asked to identify the lesions. Each ANM and staff nurse performed a VIA test, clinical breast examination, and oral examination and taught breast self-examination to women. Accredited social health activists did the motivation part for oral and breast examination through role play. Male health workers performed oral examinations on patients attending the PHC. ANMs, staff nurses, and male health workers filled out clinical forms to ensure that all the parameters were understood without any difficulty when filling out the form during screening. An ECHO session was conducted at Gumballi PHC to demonstrate how the telementoring aspect of the program would continue for further training on the ECHO platform. Positive outcomes and shortcomings of the training were discussed. After training, the same evaluation questionnaire was given to the participants of the training program to gauge the knowledge gained through training.

**Fig 1:**
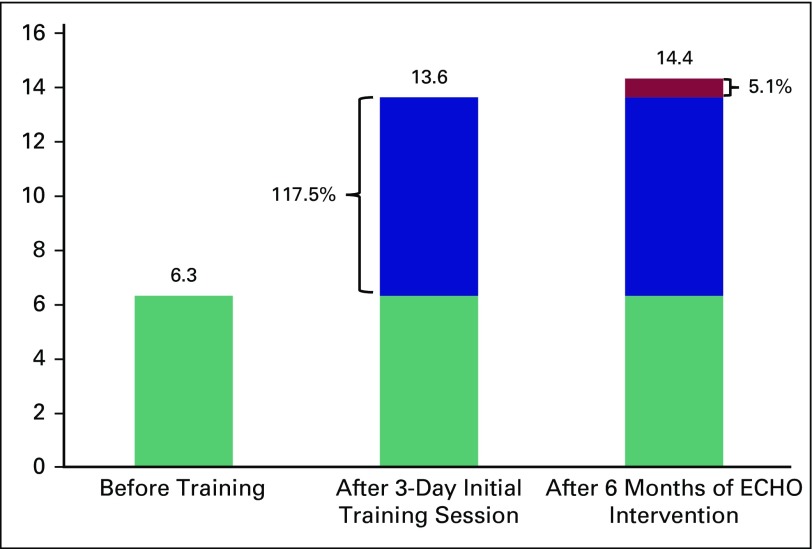
Graphical representation of knowledge scores before training, after the 3-day initial training session, and after 6 months of Extension for Community Health Outcome (ECHO) intervention. The mean scores before training (6.3), after the 3-day initial training session (13.7), and after 6 months of ECHO intervention (14.4) demonstrates the marked increase in knowledge levels immediately after training (117.5%) and the further increase in knowledge with continued virtual training on the ECHO platform (5.1%). In addition, there was no decrease in knowledge levels in any of the topic sections.

After the initial 3-day training session, the training continued every 2 weeks on the ECHO platform. ECHO sessions were scheduled on the days when the HCPs visited the PHC for their weekly meeting. These sessions were conducted in the afternoon, when the HCPs had completed their field visits, so that they did not interrupt their routine work schedule. The PHC was equipped to conduct ECHO sessions, with a desktop computer, Web camera, microphone, speakers, and broadband Internet connectivity. During these sessions, all the topics discussed during the in-person training were reiterated. In addition, any new challenges encountered by the HCPs while implementing cancer screening in the population were discussed in detail. In particular, the challenges faced by the HCPs in implementing cervical cancer screening were addressed during these virtual sessions. A total of 10 ECHO sessions were conducted. Six months after the initial training and after 10 ECHO sessions, an evaluation was conducted using the same questionnaire that was used for before and after the 3-day training session (Fig 1). This evaluation was answered by 18 HCPs who attended the PHC on that day.

### Statistical Analysis

The questionnaire consisted of 15 questions on five topics; three questions pertained to general information about cancer, seven questions were related to cervical cancer, three questions were related to breast cancer, and one question each pertained to oral cancer and sterilization of instruments. Each question had five choices with one right answer. Every right answer was given a score of 1 point, whereas wrong answers and unattempted answers were given a score of 0. Scores for each person were calculated according to the topic of question, as discussed earlier.

Univariate analysis was performed to assess the significance of various determinants. Data were analyzed using SPSS software version 21 (SPSS, Chicago, IL), and percent increase was calculated using the formula. The graph was prepared in Excel (Microsoft). Frequency distribution tables were generated. The number of questions asked per topic amounted to the highest score that could be allocated to that topic. Average scores were calculated.

## RESULTS

Table 2 lists the average scores before and after training and the maximum score (ranging from 1 to 7) for each topic. The greatest knowledge increase was seen in sterilization methods (220%), followed by cervical cancer information (194.7%) and breast cancer information (107.7%).

**Table 2:**
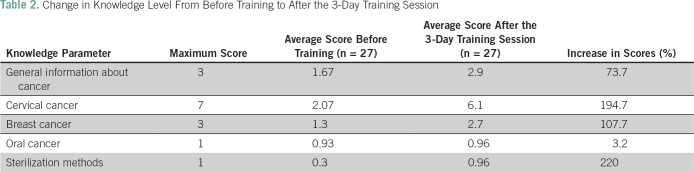
Change in Knowledge Level From Before Training to After the 3-Day Training Session

We compared the change in the knowledge level between the post-training questionnaire and the questionnaire given after 6 months of intervention (Table 3). In this comparison, the greatest increase in knowledge was seen in the areas of breast cancer (8.9%), cervical cancer (6.7%), oral cancer (4.2%), and sterilization methods (4.2%).

**Table 3:**
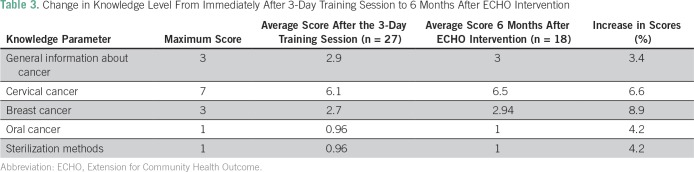
Change in Knowledge Level From Immediately After 3-Day Training Session to 6 Months After ECHO Intervention

## DISCUSSION

Our study demonstrates that the knowledge level of HCPs can be improved by training in cancer screening, which can then be effectively sustained using an economical telementoring model such as ECHO for the much needed iterative training. To the best of our knowledge, this is the first study that has used a virtual training platform like ECHO to retain the knowledge of HCPs after training.

ECHO has proven to be an effective tool to mentor and continue to train HCPs while they implement cancer screening in the community and to help troubleshoot any challenges they face in the field. ECHO is cost effective because it is on a virtual platform, thus eliminating the costs of travel for training, including resource persons, food, accommodations, and venue rentals, in addition to the costs of time taken off to travel to and attend such sessions. The total cost of setting up ECHO at a PHC includes only the additional cost of a good broadband or wireless Internet connection to an already existing desktop computer, which is available at all PHCs. These training sessions were conducted over ≤ 1 hour when the HCPs came to the PHC to attend their weekly meeting. Most importantly, ECHO eliminates travel time of the HCPs—time that can be used in screening the population. It also helps in reiteration of the study material and to update the cohort of any recent research development, which HCPs can then apply in the field to enhance the quality of cancer screening without having to wait for the next in-person training session.

ECHO is a low-cost tool that can be easily accessed on a desktop or laptop computer, tablet, or smartphone with broadband or wireless Internet connectivity, and most PHCs are equipped with a desktop computer. ECHO can be set up by adding a Web camera, microphone, and speakers at nominal cost. Many HCPs also carry smartphones with them, through which they will be able to download the software required for ECHO, which is free of cost. With the improvements in Internet connectivity and the drastic reductions in cost of Internet packages in India, this tool can be easily accessed by HCPs working in remote areas, if needed. Often, subcenters are at a significant distance from PHCs or training centers. It is a challenge for HCPs to take time off from their busy work schedule for training that is organized at the district or state level at the respective capitals. If conducted on the ECHO platform, such training will prove to be beneficial because it can save cost, time, and effort on the part of trainers and trainees. In addition, ECHO clinics allow joint problem solving to develop practical solutions for implementation challenges in the field.

The ECHO model enables trainers to convey the latest updates in research in the field to the distant HCPs, who would never be able to access such information on their own. Guiding the HCPs through the cancer screening process and troubleshooting any issues while implementing the cancers screening program will ensure high-quality screening, which is truly needed in resource-constrained areas.

One of the limitations of our study is that we do not have a control group with which to compare the results. However, no such cancer screening training was conducted for HCPs in the state until our training program. We conducted this study in only one PHC because it was a pilot program. On the basis of the success of this program, we intend to carry out a similar training program in multiple PHCs around the country. The other limitation is the participation of only 18 HCPs at 6 months after intervention evaluation. This was a result of various causes including competing priorities such as vaccination programs being conducted by the state government. Although ECHO is an effective model for iterative training, the initial in-person training is vital and cannot be eliminated.

In-person training is essential before HCPs implement a population-based cancer screening program because their knowledge about cancer and screening tests is minimal. The ECHO model is effective for continued training and reiteration of knowledge to accomplish effective population-based cancer screening in India. Using a widely spoken language, in this case Kannada, as the medium for teaching and conducting the training plays a huge role in the HCPs’ understanding of the information being taught.
